# Supplementary material for: MetaRibo-Seq measures translation in microbiomes
Source: Nat Commun. 2020 Jun 29;11:3268. doi: 10.1038/s41467-020-17081-z (PMC7324362; doi:10.1038/s41467-020-17081-z)
Supplement: Supplementary file 10 — Supplementary Data 7 [file 41467_2020_17081_MOESM10_ESM.zip › File2/Confidence_High_Taxonomy/108061_out.krona.html]

Javascript must be enabled to view this page.

members
magnitude
magnitudeUnassigned
count
unassigned
taxon
rank

108061\_out

3

1

SRS022609\_contig\_number\_2223

superkingdom
2
2

1239
2
phylum

1
class
186801

186802
order
1

family
1
216572

459786
1
genus

species
1

SRS011134\_contig\_number\_36093
1945593


SRS143342\_contig\_number\_17974
1263007
1
species
